# Patients Who Share Transparent Visit Notes With Others: Characteristics, Risks, and Benefits

**DOI:** 10.2196/jmir.3363

**Published:** 2014-11-12

**Authors:** Sara L Jackson, Roanne Mejilla, Jonathan D Darer, Natalia V Oster, James D Ralston, Suzanne G Leveille, Jan Walker, Tom Delbanco, Joann G Elmore

**Affiliations:** ^1^University of WashingtonDivision of General Internal MedicineSeattle, WAUnited States; ^2^Beth Israel Deaconess Medical Center, Harvard Medical SchoolBoston, MAUnited States; ^3^Geisinger Health SystemDanville, PAUnited States; ^4^Group Health Research Institute, Group Health CooperativeSeattle, WAUnited States; ^5^College of Nursing and Health SciencesUniversity of MassachusettsBoston, MAUnited States

**Keywords:** open access to information, caregivers, health behavior, information sharing

## Abstract

**Background:**

Inviting patients to read their primary care visit notes may improve communication and help them engage more actively in their health care. Little is known about how patients will use the opportunity to share their visit notes with family members or caregivers, or what the benefits might be.

**Objective:**

Our goal was to evaluate the characteristics of patients who reported sharing their visit notes during the course of the study, including their views on associated benefits and risks.

**Methods:**

The OpenNotes study invited patients to access their primary care providers’ visit notes in Massachusetts, Pennsylvania, and Washington. Pre- and post-intervention surveys assessed patient demographics, standardized measures of patient-doctor communication, sharing of visit notes with others during the study, and specific health behaviors reflecting the potential benefits and risks of offering patients easy access to their visit notes.

**Results:**

More than half (55.43%, 2503/4516) of the participants who reported viewing at least one visit note would like the option of letting family members or friends have their own Web access to their visit notes, and 21.70% (980/4516) reported sharing their visit notes with someone during the study year. Men, and those retired or unable to work, were significantly more likely to share visit notes, and those sharing were neither more nor less concerned about their privacy than were non-sharers. Compared to participants who did not share clinic notes, those who shared were more likely to report taking better care of themselves and taking their medications as prescribed, after adjustment for age, gender, employment status, and study site.

**Conclusions:**

One in five OpenNotes patients shared a visit note with someone, and those sharing Web access to their visit notes reported better adherence to self-care and medications. As health information technology systems increase patients’ ability to access their medical records, facilitating access to caregivers may improve perceived health behaviors and outcomes.

## Introduction

Patients are often cared for in the home by informal caregivers. In the United States, approximately 39% of adults are caregivers for an adult or child with significant health issues [1]. The care provided by these informal caregivers is valued at US $196 billion per year, in contrast to a cost nationally of US $32 billion for formal home health care, and US $83 billion for nursing homes [2]. As Americans age, with more patients with comorbid complex conditions and the prevalence of dementia likely to triple by 2050 [3,4], provision of care by family members and other close relations will likely become increasingly common. Caregivers will be called on to assist with improving quality and containing health care costs.

OpenNotes is an initiative that gives patients access to the visit notes written by their doctors, nurses, or other clinicians. In the OpenNotes study, one third of patients with easy Internet access to their primary care doctors’ notes were concerned about privacy [5]; however, they were not deterred from accessing notes and reported feeling more in control of their health care and being better able to care for themselves [6]. Access to provider documentation serves as a reminder of the clinical interaction and potentially enhances patients’ engagement with the plan of care and therapeutic regimens [6]. Additionally, providing access to paper visit notes [7,8] and electronic medical records may improve patient outcomes [9,10], making open access to visit notes a compelling intervention.

Health policy encourages patient access to their electronic health information. The Medicare and Medicaid Electronic Health Care Record (EHR) Incentive Program provides incentive payments to eligible professionals and hospitals as they adopt, implement, or show meaningful use of certified EHR technology. Stage 2 of Meaningful Use advocates electronic engagement of patients and their families [11]. A core requirement is to provide patients with electronic access to portions of their medical record, specifically after visit summaries [12]. Informal caregivers are incorporated into a framework for evaluation of the patient portal, My Health*e*Vet, used by the Department of Veteran’s Affairs [13]. However, the focus of policy and research interventions to date has primarily been on providing individual patients access to their own health information: the impact of sharing electronic records with informal caregivers is largely unknown.

Large medical systems, including the Veterans Administration (VA), Geisinger Health System, Mayo Clinic, Beth Israel Deaconess network, and MD Anderson Cancer Center are offering open records to increasing numbers of patients, and in some circumstances also to their delegates [6,14]. Prior patient surveys suggest interest in shared electronic medical records, including low income populations [15] and veterans [16]. My Health*e*Vet participants indicated that 79% of the predominantly elderly, male population was interested in sharing access to their electronic medical record laboratory results and medication refill information with someone else, most commonly with their spouse or partner [16]. In a single-site qualitative study of veterans and their delegates who had Internet access to clinical data, including visit notes, participants noted predominantly positive experiences [17], but no prior studies have focused on how patients share their visit notes with others outside of the patient-doctor dyad.

Decreasing barriers to communication of important medical information and recommendations with caregivers has the potential to improve patient outcomes and decrease medical errors. The OpenNotes study invited patients in three US states to access their visit notes for one year. Here we evaluate the characteristics of patients who reported sharing their visit notes during the course of the study, including their views on associated benefits and risks.

## Methods

### Overview

The study was a quasi-experimental intervention that invited patients to access their primary providers’ visit notes via Web-based, secure patient portals for a year between the summer of 2010 and fall of 2011. Study populations included urban and suburban primary care practices associated with Beth Israel Deaconess Medical Center (BIDMC), an academic health center with urban and suburban practices, Geisinger Health System (GHS) in central, largely rural Pennsylvania, and Harborview Medical Center (HMC), an urban safety-net teaching medical center affiliated with the University of Washington. At HMC, both the general medical clinic and a primary care clinic for patients with human immunodeficiency virus (HIV) participated in the study. Patients were initially surveyed about their expectations at the time of enrollment, and a year later surveys focused on their experiences with the intervention. The details of the study are previously described [18].

### Protection of Human Subjects

All study procedures were approved by the institutional review boards of BIDMC, GHS, and the University of Washington.

### Study Population and Survey Content

Patients of volunteering primary care physicians were invited electronically to participate at BIDMC and GHS, where portals already existed, and were approached individually at HMC, where an existing electronic health record was modified and made available to study participants [19].

Pre-intervention baseline surveys assessed patients’ demographic data, including education, self-reported health, how the patients felt about gaining electronic access to visit notes [19]. It also measured patient-doctor communication using the Ambulatory Care Experiences Survey score of patient-doctor interactions (range of 0-6 with a higher score indicating better communication), and the Perceived Efficacy of Patient-Physician Interactions score, which assesses the patient’s level of confidence communicating with their physician (range 5-50 with a higher score indicating greater confidence) [20,21]. The post-intervention survey asked participants about sharing their notes: “Did you show or discuss your visit notes with other people?” and “With whom did you share or discuss the note? (Check all that apply)”. Participants also responded to statements about the results of reading their visit notes: “I take better care of myself”, “I do better with taking my medications as prescribed”, “I am concerned about my privacy”, with response options including disagree, somewhat disagree, somewhat agree, agree, and don’t know. The full pre- and post-test surveys are available on the OpenNotes website [22].

### Statistical Analysis

Data from the baseline and post-intervention survey were analyzed for participants who viewed at least one visit note and responded “yes” or “no” to the sharing question on the post-intervention survey. The proportion of participants reporting that they showed or discussed their note with someone else during the study (sharers) was compared to those who did not (non-sharers). Sharers and non-sharers were compared by patient characteristics from the baseline survey, including demographics, self-reported health, patient-doctor communication measures, and from the post-intervention data, including number of notes available during the study, frequency of portal use, and behavioral perceptions (better self-care, better adherence to medications, concern about privacy) using chi-square tests and Mann Whitney tests when appropriate.

Modified Poisson regression with robust error variance was used to determine perceived relative risks of sharing notes for each of the aforementioned patient characteristics in univariate models. Characteristics were found statistically to be significantly associated with sharing visit notes were then included in multivariable models. The resulting characteristics associated significantly with sharing visit notes: age, gender, employment status, and study site were incorporated into relative risk models to assess the association between sharing, frequency of portal access, and behavioral perceptions, respectively. Data analyses were performed using SAS software, version 9.3.

## Results

Across the three study sites, 22,703 patients were invited to participate, 19,371 (85.32%) completed the intervention, and 11,155 of those (57.59%) had at least one note available during the study period [18]. Of those with at least one note available, 4516 (40.48%) completed the post-intervention survey and responded “yes” or “no” to the sharing survey question.

Over half (55.43%, 2503/4516) of post-intervention survey respondents agreed that they would like the option of letting family members or friends have their own access to their visit notes. In fact, 21.70% (980/4516) of participants reported showing or discussing their visit note with someone else. Among those that shared their visit notes, the persons with whom they shared included (the survey allowed reporting of more than one individual) a family member, friend, or relative who takes care of them (349/980, 35.61%), another family member (554/980, 56.53%), another friend (95/980, 9.69%), another doctor (87/980, 8.88%), a nurse or health professional (83/980, 8.47%), or someone else (107/980, 10.92%).

Multiple characteristics were significantly associated with sharing visit notes during the intervention in unadjusted analyses: being 60 years of age and older, male, having less than or equal to a high school education, being retired or unable to work, having poor or fair self-reported health, and participating at a study site other than BIDMC (Tables 1 and 2). In unadjusted analyses, those who shared were more likely to respond affirmatively to taking better care of themselves and doing better with taking their medication as prescribed and were neither more nor less likely to report concern about their privacy than non-sharers (Table 3). The median number of days that the portal was accessed during the study was 30 for those who shared their notes, compared to 28 for non-sharers. Both sharers and non-sharers had a median of three visit notes available during the study.

**Table 1:** Characteristics of patients who reported sharing or did not report sharing their visit notes with someone else during the study.

Patient characteristics^a^			Did share visit notes		Did not share visit notes		*P* value^b^
			n	%	n	%	
Total number of participants (N=4516)			980		3536		
**Age at baseline**							
	18-39		97	9.9	488	13.8	<.001
	40-49		154	15.7	691	19.5	
	50-59		284	29.0	1189	33.6	
	60-69		292	29.8	849	24.0	
	≥70		153	15.6	319	9.0	
**Gender**							
	Women		466	47.6	2256	63.8	<.001
	Men		514	52.5	1280	36.2	
**Race**							
	White		758	77.4	2781	78.7	.41
	Black or African American		15	1.5	78	2.2	
	Other or multiracial		50	5.1	169	4.8	
	Unknown		157	16.0	508	14.4	
**Education**							
	High school/GED or less		158	16.1	429	12.1	<.001
	Some college		181	18.5	616	17.4	
	College graduate		106	10.8	543	15.4	
	Post college		257	26.2	1047	29.6	
	Unknown		278	28.4	901	25.5	
**Employed**							
	No (Retired/unable to work)		311	31.7	746	21.1	<.001
	Yes (Employed/self-employed/homemaker)		374	38.2	1843	52.1	
	Unknown		295	30.1	947	26.8	
**Self-reported health**							
	Poor/Fair		118	12.0	323	9.1	.001
	Good/Very Good		523	53.4	2005	56.7	
	Excellent		61	6.2	308	8.7	
	Unknown		278	28.4	900	25.4	
**Study site**							
	**Harborview**						
		HIV clinic	21	2.1	33	<1	<.001
		Adult medicine clinic	10	1.0	12	<1	
	GHS		460	49.9	1567	54.4	
	BIDMC		489	46.9	1924	44.3	

^a^Patient characteristics were obtained from the pre-intervention survey, response rate 51.90% (5789/11,155).

^b^ *P* values derived from chi-square tests unless otherwise noted.

**Table 2:** Patient-doctor interaction and patient confidence in communication with doctor scores for patients who did share or did not share visit notes with someone else during the study.

Measures of interaction	Did share visit notes		Did not share visit notes		*P* value
	Mean (SD)	Median (IQR)	Mean (SD)	Median (IQR)	
Ambulatory care experiences survey score^a^	5.2 (0.9)	5.6 (4.8, 6.0)	5.1 (0.9)	5.4 (4.6, 5.8)	.009^b^
Perceived efficacy of patient-doctor interactions score^c^	42 (7)	44 (23, 24)	42 (7)	43 (23, 24)	.42^b^

^a^Patient report of patient-doctor interactions; range of 0-6, with a higher score indicating better communication.

^b^ *P* value obtained from Mann-Whitney test.

^c^Patient level of confidence about communicating with his or her physician; range of 5-50, with a higher score indicating more confidence.

**Table 3:** Behavioral perceptions of patients who reported sharing their visit notes with someone else during the study (N=4516).

Behavioral perceptions			Did share visit notes (n=980)		Did not share (n=3536)		*P* value^a^
			n	%	n	%	
In thinking about what it was like to read your doctor’s visit notes							
	**I take better care of myself**						
		Agree/somewhat agree	843	86.02	2737	77.40	<.001
		Disagree/somewhat disagree/don’t know	137	13.98	799	22.60	
	**I do better with taking my medications as prescribed**						
		Agree/somewhat agree	698	71.22	2103	59.47	<.001
		Disagree/somewhat disagree/don’t know	203	20.71	1058	29.92	
		Do not take medications	79	8.06	375	10.61	
	**I am concerned about my privacy**						
		Agree/somewhat agree	347	35.41	1345	38.04	.13
		Disagree/somewhat disagree/don’t know	633	64.59	2191	61.96	

^a^ *P* values derived from chi-square tests.

When demographic, health, and study site characteristics were placed into a multiple adjusted regression model, the characteristics that remained independently associated with sharing visit notes were being male, being retired or unable to work, and attending the general medicine clinic at Harborview Medical Center, an urban safety-net hospital (Table 4). After adjusting for age, gender, employment, and study site, the probability of sharing increased by 4% for each visit note available during the study (RR 1.04, 95% CI 1.03-1.06) (data not shown). After adjustment for the same demographic and study site characteristics, participants who shared were statistically significantly more likely to report taking better care of themselves (RR 1.45, 95% CI 1.20-1.76) and taking their medication as prescribed (RR 1.49, 95% CI 1.25-1.76), they were no more or less concerned about their privacy than were non-sharers (Table 5).

**Table 4:** Unadjusted and adjusted association between patients who shared visit notes and demographic characteristics, self-reported health, and study site.

Characteristic			Unadjusted RR^a,b^	95% CI	Adjusted RR^a,b^	95% CI
**Age at baseline**						
	18-39		1		1	
	40-49		1.10	0.87-1.38	0.96	0.72-1.28
	50-59		1.16	0.94-1.43	0.97	0.75-1.26
	60-69		1.54	1.25-1.90	1.09	0.83-1.44
	≥70		1.95	1.56-2.44	1.22	0.89-1.66
**Gender**						
	Men		1.67	1.50-1.87	1.61	1.41-1.85
	Women		1		1	
**Education**						
	High school/GED or less		1.37	1.15-1.62	1.19	0.97-1.46
	Some college		1.15	0.97-1.36	1.10	0.92-1.32
	College graduate		0.83	0.67-1.02	0.86	0.70-1.05
	Post college		1		1	
**Employment**						
	No (Retired/unable to work)		1.74	1.53-1.99	1.39	1.18-1.64
	Yes (Employed/homemaker)		1		1	
**Self-reported health**						
	Poor/Fair		1		1	
	Good/Very good		0.77	0.65-0.92	0.88	0.74-1.05
	Excellent		0.62	0.47-0.81	0.78	0.59-1.04
**Study site**						
	**Harborview**					
		HIV clinic	1.92	1.36-2.71	1.35	0.90-2.01
		Adult medicine clinic	2.24	1.41-3.57	1.67	1.01-2.76
	GHS		1.12	1.00-1.25	0.96	0.82-1.13
	BIDMC		1		1	

^a^Estimates derived from modified Poisson regression with robust error variance.

^b^Adjusted for age, gender, education, employment, self-reported health and study site.

**Table 5:** Behavioral perceptions in patients who shared visit notes.

Behavioral perceptions		Unadjusted RR^a^	95% CI	Adjusted RR^a,b^	96% CI
**In thinking about what it was like to read your doctor’s visit notes**					
	I take better care of myself	1.61	1.36-1.90	1.45	1.20-1.76
	I do better with taking my medications as prescribed	1.55	1.34-1.78	1.49	1.25-1.76
	I am concerned about my privacy	0.91	0.81-1.03	0.94	0.82-1.08

^a^Estimates derived from modified Poisson regression with robust error variance.

^b^Adjusted for age, gender, employment, and study site.

## Discussion

### Principal Findings

Health care systems, prodded by policy drivers and consumer demand, are increasingly moving forward with opening records to patients [6,18,25], but how these records are shared with others is not well understood. In this study of open access to doctors’ visit notes in three disparate outpatient settings, one-fifth of participants reported sharing their notes with a variety of individuals over the course of a year, and 55% reported interest in allowing family or friends their own access to their visit notes. This potentially indicates that with a longer study duration, more patients might have shared notes or shared with people who are geographically distant. Those who shared were more likely to report taking better care of themselves and taking their medications as prescribed. They were also more likely to be older, male, less educated, unemployed, and have poorer self-reported health than were those who chose not to share. While a sizable minority of patients surveyed expressed concerns about loss of privacy, we found no difference in such worries between the sharers and those choosing to keep notes to themselves.

A large proportion of persons in the United States are informal caregivers [1,26], and caregiver support groups encourage them to take notes and actively participate in doctors’ visits, including accessing patient information via portals [27]. “Care partners” who do not provide day-to-day care but do help patients navigate the health care system and facilitate communication with providers, sometimes from a distance, would also benefit from access to online patient portals [28]. However, formal study of patients’ and caregivers’ desires around sharing notes is limited. Data from a predominantly older, male Veteran population suggest that their interest in sharing medical records is high [16]. In the OpenNotes study, conducted among a younger, more diverse population with a larger number of female patients, the majority of participants similarly desired the option to share notes. As more health care institutions offer access to patient records and notes, it is likely that more and more patients will share their health information with others.

Integrating caregivers of frail patients into each step of patient care will likely become increasingly important for providing high-quality and cost-effective care for these medically complex patients [29]. Nearly half of caregivers perform tasks typically carried out by professional health care workers, such as wound care or intravenous medication administration [30]. Similarly caregivers for the burgeoning number of patients with dementia [3] often need to acquire skills of professional medical personnel. For all members of a medical team, rapid and open communication can be vital, and the visit note can serve as an expedient and effective platform. It should help both patients and their caregivers engage, communicate, develop and demonstrate trust, and assist with the implementation of the care plan. Further investigation regarding the interaction within patient-caregiver dyads has substantial implications for the safe and effective implementation of patient portals and the requirements that health information technology vendors need to consider in the development of this technology.

Accessing medical information via the Internet has been postulated to increase the “digital divide” between those who are facile with technology and those who are not [31]. Patients who reported improved health behaviors after the OpenNotes study were more likely to have shared their notes. Given this, it is noteworthy that the patients attending the Harborview clinics, which have a specific mission to care for the community’s most vulnerable patients, were more likely to have shared their notes than those from the other clinical sites. Moreover, older and unemployed patients, and those who reported poorer health were also more likely to do so. This suggests that those with greater medical comorbidity and potentially less health literacy may value and benefit most from the ability to share their notes by seeking help from support persons. As patients turn to caregivers for help, the opportunity for patients to share their notes may help diminish a digital divide.

Clinicians often record intimate details of patients’ lives. Electronic access to such information for persons outside of the patient-doctor relationship raises concerns about privacy. Substance use, mental health, and sexual history, for example, are areas that many patients could be reticent to share with caregivers. However, we found that those who shared their visit notes were neither more nor less concerned about privacy than were non-sharers. While this is reassuring for those who advocate open access to patient notes, perhaps the proportion of those who shared would have been greater if elements of the “social history” were restricted. Health information technology vendors need to consider how to protect patients’ privacy while facilitating access to pertinent medical information and recommendations. Currently there are no clear standards for caregiver access to patient portals, and authentication procedures vary widely [28]. Additional study of how to document sensitive personal history, while potentially allowing patients to not disclose the social history to caregivers or family, would benefit patients and caregivers.

Caregiver stress is well documented [2]. While access to the patient’s visit notes may decrease stress by facilitating communication and clarifying the plan, it could also induce stress. The chasm between a caregiver’s experience of the patient’s illness and the physician’s understanding of the diagnosis and science may trigger tension between caregiver and doctor [32], and how concepts are communicated in a visit note could alienate the caregiver. Content within an electronic medical record, which frequently includes both data and repetition designed primarily for billing and administrative purposes, as well as medical jargon and speculative diagnostic scenarios that could include frightening diagnostic possibilities, may in itself increase caregiver stress. In the OpenNotes study, some physicians reported changing the way they phrased potentially sensitive information related to malignancy, mental health, or substance use [18]. Viewing both patients and caregivers as integral members of the health care team, doctors and EHR vendors should maximize clear, direct communication in visit notes, and hopefully diminish stress and anxiety that caregivers might feel.

As patients share notes with caregivers, they may also consider sharing their notes more broadly, such as posting them on social media platforms. Divulging sensitive information, wittingly or unwittingly, could affect personal relationships, job opportunities, or litigation. A doctor’s note freely accessible on the Internet could generate positive or negative comment from a wide variety of viewers. As social media evolve hand in hand with health care transparency, the consequences for the doctor-patient relationship are largely unknown, and adding caregivers to the mix may introduce even more complexity.

### Strengths and Limitations

This study’s strengths include the large number of patients granted access to their visit notes in geographically and socioeconomically diverse settings. These participants may represent early adopters of technology that may quickly become standard of practice. But limitations derive from a sample that nevertheless represents a small subset of Americans, so these findings cannot be considered widely generalizable. The data are self-reported survey data, without input from caregivers.

### Conclusions

We have undergone a revolution in the way personal electronic data are accessed and shared. Future patient portals will need to integrate the preferences of patients, caregivers, and health care providers. Developing separate secure portals for caregivers may help limit access to components that the patient prefers to keep private. Vendors will need to add features allowing patients to share specific information with caregivers based on patient preferences. Policies for organizations seeking to enable “delegation” for patients are needed to address aspects such as authentication of patient delegates and how control of specific access to patient information is supported. Protections against inadvertent over-sharing must also be considered.

Coordinating care for patients is both a tremendous challenge and a core competency for effective care organizations [33]. When given the opportunity, 22% of OpenNotes patients shared their visit notes with someone over a year’s time. Those who shared reported that they took better care of themselves, were more likely to take their medications, and were not more or less concerned about privacy than non-sharers. Sharing visit notes to engage caregivers and family as active members of the health care team could be critical for many patients if clinical outcomes are to improve and costs are to be contained. Open access to visit notes offers exciting opportunities to engage a patient’s family and social support members, and now is the time to establish standards and develop the technology to open these portals.

## Abbreviations

BIDMC: Beth Israel Deaconess Medical Center
EHR: Electronic Health Records
GED: General Educational Development
GHS: Geisinger Health System
HIV: human immunodeficiency virus
HMC: Harborview Medical Center
VA: Veterans Administration
